# Pharmacokinetics of Orally Applied Cannabinoids and Medical Marijuana Extracts in Mouse Nervous Tissue and Plasma: Relevance for Pain Treatment

**DOI:** 10.3390/pharmaceutics15030853

**Published:** 2023-03-06

**Authors:** Cristiana Dumbraveanu, Katharina Strommer, Meinolf Wonnemann, Jeiny Luna Choconta, Astrid Neumann, Michaela Kress, Theodora Kalpachidou, Kai K. Kummer

**Affiliations:** 1 Institute of Physiology, Medical University of Innsbruck, 6020 Innsbruck, Austria; 2 Bionorica Research GmbH, 6020 Innsbruck, Austria; 3 Independent Researcher, 92318 Neumarkt, Germany

**Keywords:** medical marijuana, THC, tetrahydrocannabinol, CBD, cannabidiol, bioavailability, neuropathic pain, spared nerve injury

## Abstract

Cannabis sativa plants contain a multitude of bioactive substances, which show broad variability between different plant strains. Of the more than a hundred naturally occurring phytocannabinoids, Δ9-Tetrahydrocannabinol (Δ9-THC) and cannabidiol (CBD) have been the most extensively studied, but whether and how the lesser investigated compounds in plant extracts affect bioavailability or biological effects of Δ9-THC or CBD is not known. We therefore performed a first pilot study to assess THC concentrations in plasma, spinal cord and brain after oral administration of THC compared to medical marijuana extracts rich in THC or depleted of THC. Δ9-THC levels were higher in mice receiving the THC-rich extract. Surprisingly, only orally applied CBD but not THC alleviated mechanical hypersensitivity in the mouse spared nerve injury model, favoring CBD as an analgesic compound for which fewer unwanted psychoactive effects are to be expected.

## 1. Introduction

Cannabinoids are bioactive substances found in the *Cannabis sativa* plants, which show broad variability between different cannabis strains [1,2]. Of the more than a hundred naturally occurring phytocannabinoids that have been identified so far, Δ9-Tetrahydrocannabinol (Δ9-THC) and cannabidiol (CBD) are the best studied and have been shown to possess distinct physiological and therapeutic properties [3]. Cannabinoids bind to the G protein coupled (GPCR) CB1 and CB2 receptors, which are widely expressed throughout the nervous system as well as peripheral tissues [4,5], and to different orphan GPCRs, such as GPR55, GPR18, GPR3, GPR6 and GPR12, and *peroxisome proliferator activated receptor gamma* (PPARγ) [6]. In addition, CBD acts as modest affinity agonist at human 5-HT1a receptor [7,8] and transient receptor potential (TRP) channels TRPV1, TRPV2, TRPV3, TRPV4, TRPA1 and TRPM8 [9].

While THC and its metabolite 11-hydroxy-Δ9-THC (OH-THC) are the main psychoactive components of *Cannabis sativa*, CBD is considered non-psychoactive and can modulate THC-intoxicating and metabolic effects [10,11]. Due to their lipophilic chemical structures, THC and CBD can easily enter all organs including the brain, where they can be detected for more than 24 h after application [12,13,14], despite rapid first-pass hepatic metabolism [15,16].

Already in 1974, cannabis extracts were considered to provide greater pharmacological effects compared to pure cannabinoids [17], which might be due to additional bioactive substances included in extracts, such as minor phytocannabinoids, terpenes or flavonoids [18,19]. This complementary action of the full-spectrum cannabis extracts over the pure cannabinoids is evident mainly from in vitro models using adenocarcinoma cells [20], epithelial cells and colon tissue [21].

For therapeutic interventions, different cannabis preparations exert positive effects for cancer and arthritis pain, headache, as well as mental disorders, such as depression, anxiety, post-traumatic stress and sleeping disorders [22,23]. Interestingly, the combination of THC and CBD increases the anti-allodynic potency by a factor of 200 [24], whereas the combination of cannabinol (CBN) and CBD provides analgesic relief for chronic muscle pain [25]. In addition, the application of a CBD-enriched cannabis extract completely abolishes mechanical allodynia in a mouse model of neuropathic pain [26].

Despite their wide use as painkillers, data on the pharmacokinetics of cannabinoids upon oral administration are sparse, and the effects of the less abundant cannabinoids as well as flavonoids and terpenes on bioavailability, especially in the nervous tissue, are currently not available. We therefore assessed the differences in the pharmacokinetics of cannabinoids and metabolites after oral application of medical marijuana extracts as well as pure THC and CBD compounds in plasma, brain and spinal cord tissues in naive mice and evaluated their analgesic properties in a neuropathic pain mouse model.

## 2. Materials and Methods

### 2.1. Medical Marijuana Extracts and Cannabinoid Compounds

A cannabinoid-rich extract (THC+ extract) and cannabinoid-depleted extract (THC- extract) were provided by Bionorica research GmbH (Innsbruck, Austria). The THC+ extract was obtained from *Cannabis flos* by extraction with heptane and subsequent decarboxylation. For the THC- extract, the heptane-extracted *Cannabis flos* was further extracted with 50% (*v/v*) ethanol/water. Pure THC (99%) and CBD (99%) compounds were acquired from Bionorica SE (Neumarkt, Germany) and Trigal Pharma (Vienna, Austria), respectively.

### 2.2. Reference Solutions and Extract Preparation

As reference compounds, Cannabichromene (CBC), Cannabidiol (CBD), Cannabidiolic acid (CBDA), Cannabidivarin (CBDV), Cannabigerol (CBG), Cannabinol (CBN), Tetrahydrocannabivarin (THCV), Δ9-Tetrahydrocannabinolic acid A (THCA-A), and Δ9-Tetrahydrocannabinol (Δ9-THC) were obtained from Lipomed (Arlesheim, Switzerland) and flavonoids Apigenin (API), Luteolin (LUT) and the internal standard Amarogentin (AM) were obtained from Phytolab (Vestenbergsgreuth, Germany). Reference solutions were prepared to yield calibration standards by serial dilutions with 50% methanol from 10 to 1.00 × 10^4^ ng/mL.

The quantification of bioactive substances contained in medical marijuana extracts was performed by working with extract stock solutions prepared with methanol in triplicates. The THC+ extract stock solution was diluted to 400, 40 and 4 μg/mL, and the THC- extract was diluted to 400 and 40 μg/mL, with 50% (*v/v*) methanol. Calibration or extract samples were mixed with the internal standard (AM stock solution: 428.4 µg/mL in 60% acetonitrile, AM working solution: 1000 ng/mL in 50% methanol) in a 1:1 ratio. After centrifugation at 18,300× *g* for 90 s, the supernatant was transferred into amber glass vials. Then, 50% (*v/v*) methanol was used as a blank solvent.

### 2.3. LC-ToF-MS Analysis of Medical Marijuana Extracts

Cannabinoids (CBC, CBD, CBDA, CBDV, CBG, CBN, THCV, THCA-A and Δ9-THC) and flavonoids (API and LUT) were quantified in both THC+ and THC- extracts using liquid chromatography time-of-flight mass spectrometry (LC-ToF-MS). Chromatographic separation was performed with a UHPLC System (1290 Infinity series, Agilent, Germany). Each sample was injected at a volume of 2 μL into a Zorbax RRHD Eclipse C18 column (50 × 2.1 mm, 1.8 μm; Agilent, Waldbronn, Germany) at 40 °C at a flow rate of 0.6 mL/min. The mobile phase system consisted of 0.1% formic acid in water (eluent A) and acetonitrile (eluent B). For separation of the individual cannabinoid and flavonoid compounds, the following analytical gradient was employed: 5% B (0–1 min), 5–31% B (1–6 min), 31–100% B (6–10 min), 100% B (10–11 min), 100–5% B (11–11.1 min) and 5% B (11.1–13 min). Detection was performed by means of a high-resolution triple quadrupole time-of-flight mass spectrometer (Q-Tof MS; TripleToF^®^ 5600; Sciex, Framingham, MA, USA) in the negative electrospray ionisation (ESI) mode. Spectra were recorded in the m/z range of 100–1300 Da. The m/z range for the recording of MS/MS fragmentation data in the information dependent acquisition (IDA) mode was set to 50–1300 Da. Detection parameters are detailed as follows: curtain gas, 25; gas 1, 70; gas 2, 55; turbo ion spray voltage floating, −4500 V; source temperature, 500 °C. The LC-ToF-MS system was operated with the Software Analyst^®^ TF Version 1.8.1 (Sciex, Framingham, MA, USA).

### 2.4. Animals

Adult male C57BL/6J mice (8–10 weeks; Janvier Labs, Le Genest-Saint-Isle, France) were housed under specific pathogen-free (SPF) conditions. Animals were maintained in individual cages at constant room temperature (24 °C) on 12 h light/dark cycle (lights on from 07:00 to 19:00) and had *ad libitum* access to autoclaved pelleted food and water for an acclimatization phase of 7 days. All procedures involving animals were carried out in accordance with the Ethics Guidelines of Animal Care (Medical University of Innsbruck), as well as the Directive 2010/63/EU of the European Parliament and of the Council of 22 September 2010 on the protection of animals used for scientific purposes and approved by the Austrian Bundesministerium für Wissenschaft, Forschung und Wirtschaft (permit number GZ: 2020–0.432.251).

### 2.5. Administration of Cannabinoid Formulations

For bioavailability experiments, mice were food deprived overnight preceding administration of single-dose cannabinoid formulations, to control for stomach content. For behavioral experiments, mice had ad libitum access to food for the entire duration of the experiment. Cannabinoid preparations were suspended in sesame oil and stored at −20 °C. Prior to administration, the formulations were sonicated for 15 min at 30 °C. Administration of 5 μL/g cannabinoid formulation was performed by oral gavage using disposable animal feeding needles (20 G, Sigma-Aldrich, Saint Louis, MO, USA). Doses were selected based on literature reports [24,27,28].

### 2.6. Spared Nerve Injury (SNI) Model

SNI surgery was performed as previously described [29]. In short, mice were anesthetized by intraperitoneal (i.p.) injections of 10 mg/kg Xylazine (AniMedica GmbH—a LIVISTO company, Senden, Germany) and 100 mg/kg Ketamine (AniMedica GmbH—a LIVISTO company, Senden, Germany). The skin on the lateral surface of the thigh was incised and the sciatic nerve exposed by separating the biceps femoris muscle through incision of the connective tissue without wounding the muscle. The common peroneal and tibial nerves were ligated with 4-0 Vicryl (Sh-1 plus; Raritan, NJ, USA) and a portion of 3 mm length was excised around the ligation site. Care was taken to avoid any mechanical damage to the sural nerve. After dissection, the muscle and skin were sutured using 4-0 Vicryl. Mice were left to recover at 37 °C until they regained consciousness.

### 2.7. Tissue Collection and Sample Preparation

Time-points for cannabinoid administration and tissue harvesting were planned in a counter balanced design to avoid batch effects. For each formulation, three mice were sacrificed at 0.5, 1, 2, 4, and 6 h after administration. Following deep anesthesia by i.p. injection of Ketamine/Xylazine (Ketamine 100 mg/kg, Xylazine 10 mg/kg), whole blood was drawn via cardiac puncture in 0.5 M EDTA rinsed syringes. Plasma was isolated by centrifugation and collection of the supernatant (9000× *g* for 10 min at 4 °C). After cardiac puncture, mice were perfused with 1× PBS (Gibco) in order to clear brain and spinal cord tissue of remaining blood, and whole brain and spinal cord tissue samples were collected, frozen on dry ice and stored at −80 °C until further analysis.

Whole brain and spinal cord samples were weighed. Whole brain samples were minced and mixed with 70% ethanol (*v/v*) extraction solvent to obtain a final concentration of approximately 50% (*m/v*). Whole spinal cord samples were extracted with a four-fold volume extraction solvent under pulsed shaking (600 rpm) for 30 min. In total, 50 µL of plasma, spinal cord or brain homogenate were mixed with 150 μL of the internal standard working solution, Δ*9-Tetrahydrocannabinol-D_3_* (working solution 5 ng/mL in acetonitrile, (THC-D3; Lipomed, Arlesheim, Switzerland)). Following centrifugation (18,300× *g*, 1 min), the supernatant was transferred into a 96-well plate.

### 2.8. LC-MS/MS Quantification of Δ9-THC, OH-THC, COOH-THC and CBD in Tissue Samples

A total of eight tissue-free standards were prepared to obtain a calibration curve for Δ9-THC, OH-THC (Lipomed, Arlesheim, Switzerland), COOH-THC (Lipomed, Arlesheim, Switzerland) and CBD (Trigal Pharm, Vienna, Austria), with concentrations in the range of 1–500 ng/mL. Quantification of cannabinoids in tissue samples was performed by LC-MS/MS using a 1290 Infinity series UHPLC System (1290 Infinity series, Agilent, Germany) coupled to a triple quadrupole mass spectrometer (API 4000^®^, Sciex, Framingham, MA, USA). LC separation was achieved on a Nucleodur C18 Isis reverse-phase column (50 × 2.0 mm, 1.8 μm; Macherey-Nagel, Germany). The mobile phase system was 0.05% acetic acid in water (eluent A) and acetonitrile (eluent B) at a flow rate of 0.6 mL/min and 50 °C. Detection was performed in the positive electrospray ionization (ESI) mode applying the following detection parameters: curtain gas, 30; CAD gas, 6; gas 1, 50; gas 2, 60; ion spray voltage, 4500 V; source temperature, 500 °C. The LC-MS/MS system was operated with the Software Analyst^®^ Version 1.7.1 (Sciex, Framingham, MA, USA). Transitions were recorded using the multiple reaction monitoring (MRM mode, Table 1).

### 2.9. Analysis of Quantitative Mass Spectrometry Data

Concentrations calculated by mass spectrometry were rounded to four significant digits and accuracies were reported with two decimal places by the chromatographic data processing software Sciex OS/Analytics 2.0.0 or Analyst 1.7.1. Based on the peak area ratios (analyte/internal standard) of the calibration standards, a calibration curve fit was established for each analyte. A quadratic regression model with weighting factor 1/x was used. The bioanalytical method used was not fully validated. However, the performance of each analytical batch was evaluated by the quality of the calibration curve fit as well as the back-calculated concentrations of the calibration standards. Acceptable correlation coefficients (r ≥ 0.99) as well as accuracies meeting specifications of the bioanalytical method validation guideline ICH M10 were obtained (https://www.ema.europa.eu/en/ich-m10-bioanalytical-method-validation-scientific-guideline (Accessed on 16 February 2023); ±15% or ±20% for the lowest concentration level).

Further statistical calculations were performed, and figures were generated using Excel 2016 (Microsoft, Redmond, WA, USA) and Prism 9.0 (GraphPad Software, San Diego, CA, USA). Plasma, brain and spinal cord cannabinoid concentrations at each time point were averaged and the pharmacokinetic parameters were calculated by non-compartmental pharmacokinetic analysis. Pharmacokinetic profiles indicated the actual sampling time and concentration data normalized by tissue wet weight. The linear trapezoidal method was used to calculate the AUC_0–2h_ (area under concentration-time curve). Brain–plasma and spinal cord–plasma ratios were calculated based on AUCs. The level of statistical significance was predefined at *p* < 0.05.

### 2.10. Von Frey Test for Mechanical Thresholds

The von Frey test was performed as previously described [29]. In brief, mice were placed in a Plexiglas chamber on an elevated iron-mesh floor and allowed to habituate before testing. To determine paw withdrawal thresholds, a series of custom made calibrated von Frey filaments with uniform tip diameter (diameter: 1.1 mm; forces: 2.8, 4, 5.7, 8, 11.4, 16, 22.6, 32 and 45.3 mN) was presented perpendicularly to the lateral side of the plantar paw surface (sural nerve innervation territory). Withdrawal thresholds were calculated according to the up-and-down method [30,31]. Von Frey results were analysed using repeated measure ANOVA. The level of statistical significance was predefined at *p* < 0.05.

### 2.11. Data Analysis

For statistical data analyses, GraphPad Prism 9 (GraphPad Software, San Diego, CA, USA) was used. Statistical tests used are specified in the respective methods section as well as in the text or in their respective Figures or Tables. Graphs were plotted in GraphPad Prism 9 (GraphPad Software, San Diego, CA, USA) and figures prepared using CorelDRAW 2021 (Alludo, Ottawa, ON, Canada).

## 3. Results

### 3.1. Quantification of Bioactive Cannabinoids and Flavonoids in Medical Marijuana Extracts

Anticipated differences in the composition of bioactive cannabinoids in the THC+ and THC- extracts were confirmed by mass spectrometry analysis. The THC+ extract contained all measured cannabinoids except for CBDA and no flavonoids (Table 2), with the highest concentrations for Δ9-THC (51.08% [m/m]) and CBN (6.69% [m/m]). In comparison, the THC- extract was devoid of the majority of cannabinoids and only contained detectable levels of >0.01% [m/m] of THCA-A (0.53%), Δ9-THC (0.13%), CBN (0.07%), and CBG (0.014%) (Table 3).

### 3.2. Pharmacokinetic Profile of Δ9-THC Bioavailability in Nervous Tissue and Plasma after Application of Medical Marijuana Extracts and Pure Compound

**Figure 1:**
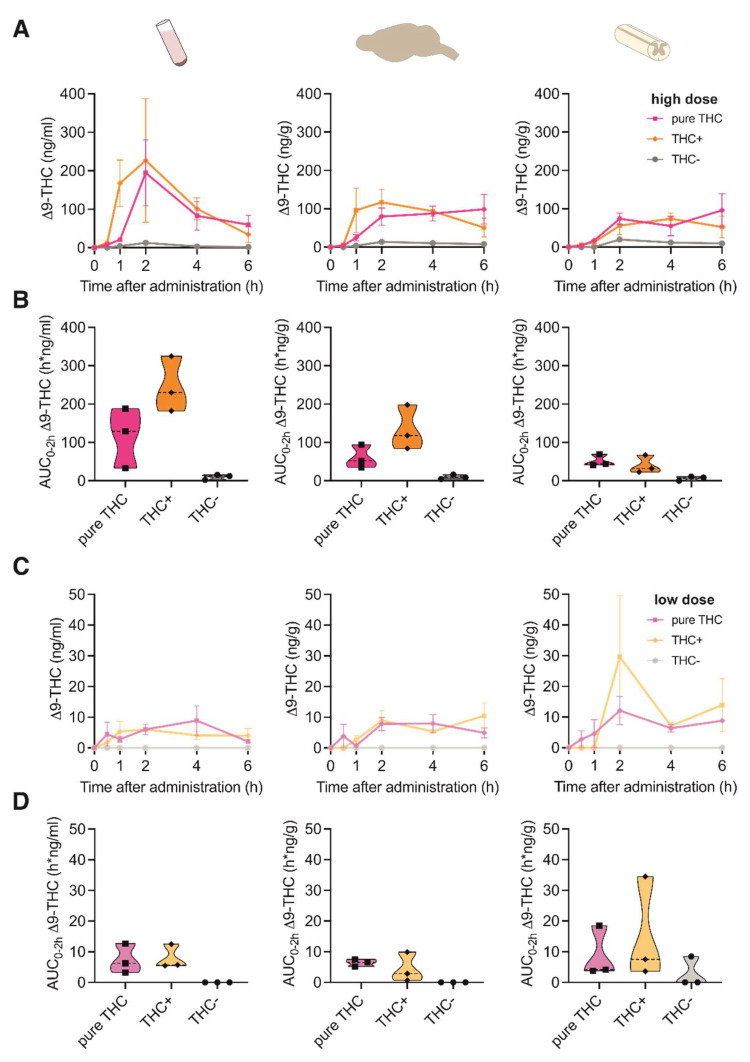
Pharmacokinetic profile of Δ9-THC following oral administration of high and low doses of cannabis extracts and pure THC compound. (**A**) Concentration-time profiles of Δ9-THC in plasma, brain and spinal cord at 0, 1, 2, 4 and 6 h after oral administration of 20 mg/kg pure THC compound, 40 mg/kg THC+ extract and 1560 mg/kg THC- extract. Data are represented as mean ± SEM, *n* = 3 per timepoint. (**B**) Area under the curve (AUC) of Δ9-THC concentrations calculated for the time period 0–2 h after administration for data shown in (**A**) (AUC_0–2h_). (**C**) Concentration-time profiles of Δ9-THC plasma, brain and spinal cord at 0, 1, 2, 4 and 6 h after oral administration of 1 mg/kg pure THC compound, 2 mg/kg THC+ extract and 40 mg/kg THC- extract. Data are represented as mean ± SEM, *n* = 3 per timepoint. (**D**) AUC_0–2 h_ of Δ9-THC concentrations calculated for data shown in (**C**).

To assess the pharmacokinetic profile of THC in tissues that are involved in the modulation of pain signaling, we determined THC concentrations in brain, spinal cord and plasma following oral gavage of two different doses (i.e., low vs. high) of THC+ (2 vs. 40 mg/kg bodyweight) and THC- extract (40 vs. 1560 mg/kg bodyweight) as well as pure THC compound (1 vs. 20 mg/kg bodyweight) at different time-points (i.e., 30 min, 1 h, 2 h, 4 h, and 6 h after gavage). Upon administration of the high doses of the THC+ extract and pure THC, Δ9-THC was detectable in all tissues 1 h after administration, reaching its maximum concentration at 2 h (Figure 1A). While Δ9-THC levels in plasma showed a decline after 2 h, they remained elevated in brain and spinal cord tissue until the last measurement. As expected, administration of the high dose of the THC- extract did not induce relevant levels of Δ9-THC in any of the investigated samples. When determining the area under the curve for the initial 2 h post administration (AUC_0–2h_), we found ~2-fold higher cumulative concentrations of Δ9-THC after application of the THC+ extract as compared to pure THC compound in both plasma and brain tissue, suggesting an improved bioavailability through more efficient absorption of Δ9-THC from the extract (Figure 1B). Administration of the low doses of the THC+ extract and pure THC only resulted in negligible concentrations of Δ9-THC in all sample types analysed (Figure 1C,D).

### 3.3. Pharmacokinetic Profile of Δ9-THC Metabolites in Nervous Tissue and Plasma after Application of Medical Marijuana Extracts and Pure Compound

We next investigated if the concentration of Δ9-THC metabolites differed between the different cannabinoid extracts or compounds in the tissues investigated. For this, we measured the concentrations of the two main secondary metabolites of Δ9-THC, 11-Nor-9-carboxy-Δ9-THC (COOH-THC) and 11-hydroxy-Δ9-THC (OH-THC). As the administration of the low dose for all formulations provided negligible concentrations for Δ9-THC, the metabolites were assessed only after administration of the high dose.

**Figure 2:**
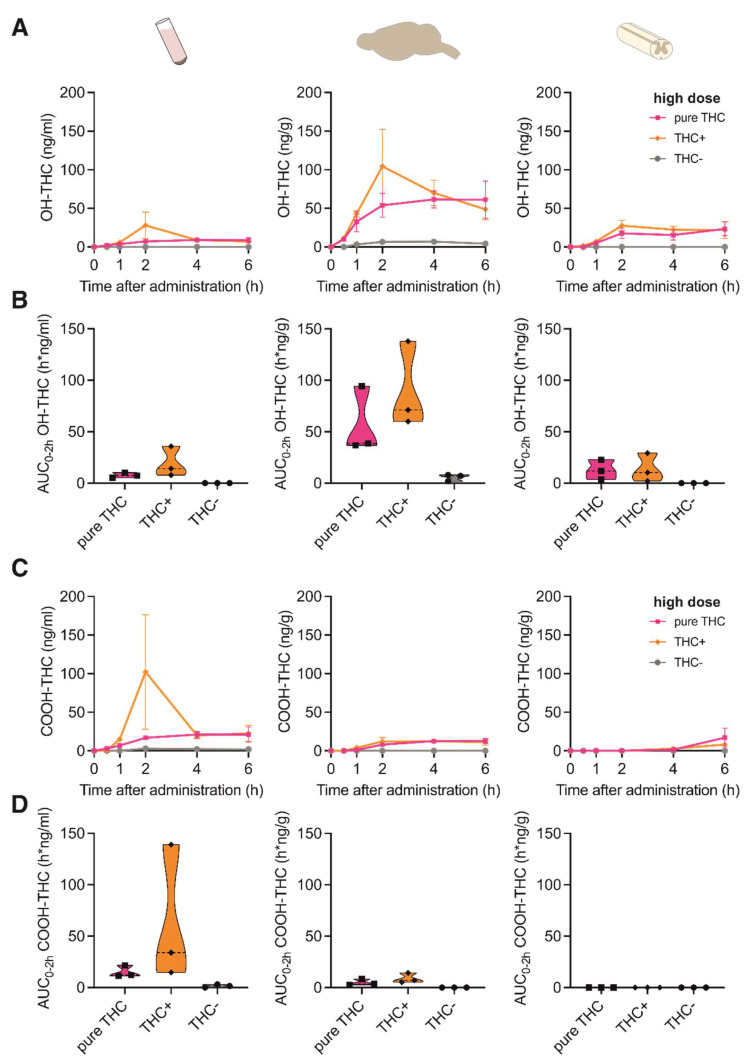
Pharmacokinetic profile of THC metabolites following oral administration of cannabis extracts and pure THC compound. (**A**) Concentration-time profiles of OH-THC in plasma, brain and spinal cord at 0, 1, 2, 4 and 6 h after oral administration of 20 mg/kg pure THC compound, 40 mg/kg THC+ extract and 1560 mg/kg THC- extract. Data are represented as mean ± SEM, *n* = 3 per timepoint. (**B**) AUC_0–2h_ of OH-THC concentrations calculated for data shown in (**A**). (**C**) Concentration-time profiles of COOH-THC in plasma, brain and spinal cord at 0, 1, 2, 4 and 6 h after oral administration of 20 mg/kg pure THC compound, 40 mg/kg THC+ extract and 1560 mg/kg THC- extract. Data are represented as mean ± SEM, *n* = 3 per timepoint. (**D**) AUC_0–2h_ of COOH-THC concentrations calculated for data shown in (**C**).

OH-THC was mainly found in brain tissue after application of both THC+ extract and pure THC compound, with a peak concentration at 2 h after application (Figure 2A) and a ~1.5-fold increased absorption after application of the THC+ extract compared with the pure THC compound (Figure 2B). In contrast, COOH-THC was found mainly in one plasma sample of the THC+ extract group (Figure 2C,D). While this sample was displaced far from the remaining samples for this time point, it survived the outlier analysis (Grubb’s outlier test, Z = 1.1433 vs. Z_critical_ = 1.1543, *p* > 0.05). These results suggest tissue-specific degradation mechanisms of Δ9-THC.

### 3.4. Pharmacokinetic Profile of CBD Bioavailability in Nervous Tissue and Plasma after Application of Medical Marijuana Extracts and Pure Compound

**Figure 3:**
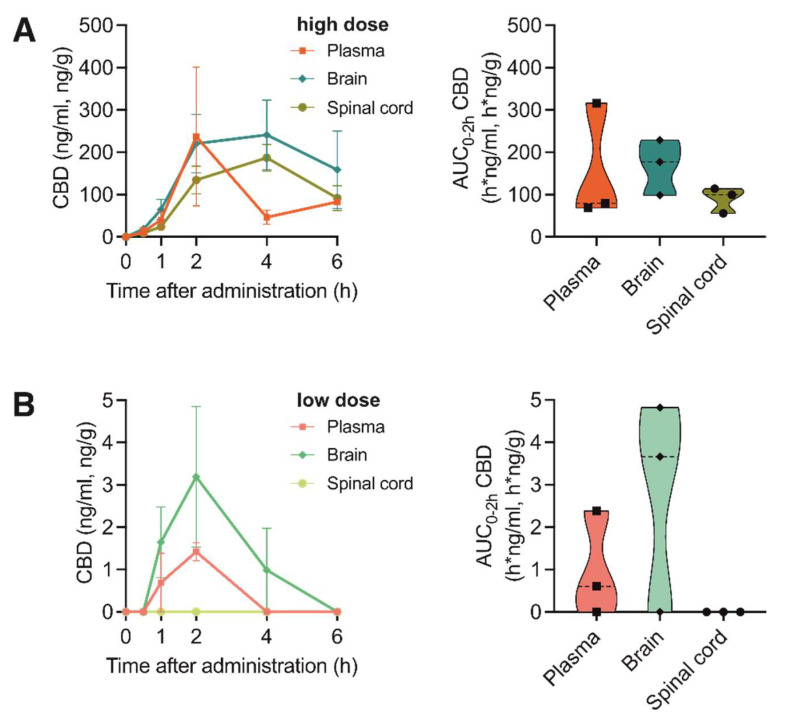
Pharmacokinetic profile of CBD after oral administration of high and low doses of pure CBD compound. (**A**) Left: Concentration-time profiles of CBD in plasma, brain and spinal cord at 0, 1, 2, 4 and 6 h after oral administration of 20 mg/kg pure CBD compound. Data are represented as mean ± SEM, *n* = 3 per timepoint. Right: AUC_0–2h_ of CBD concentrations. (**B**) Left: Concentration-time profiles of CBD in plasma, brain and spinal cord at 0, 1, 2, 4 and 6 h after oral administration of 1 mg/kg pure CBD compound. Data are represented as mean ± SEM, *n* = 3 per timepoint. Right: AUC_0–2h_ of CBD concentrations.

When evaluating the CBD bioavailability after oral application of the THC+ and THC- extract, which contained only 0.16% or non-detectable concentrations of CBD, respectively (Table 2 and Table 3), as expected, no CBD was measurable. However, application of two different doses of pure CBD compound (1 vs. 20 mg/kg bodyweight) resulted in a dose-dependent absorption of CBD reaching its maximum concentration at 2 h after application (Figure 3A,B). Interestingly, when directly comparing the absorption of pure THC and pure CBD compounds in the investigated tissues, CBD showed a 2.8- and 1.7-fold higher absorption rate compared with THC absorbance in the brain and spinal cord, respectively. This suggests a better uptake of the cannabinoid CBD in nervous tissue.

### 3.5. Analgesic Potency of Medical Marijuana Extracts and Pure Cannabinoid Compounds

**Figure 4:**
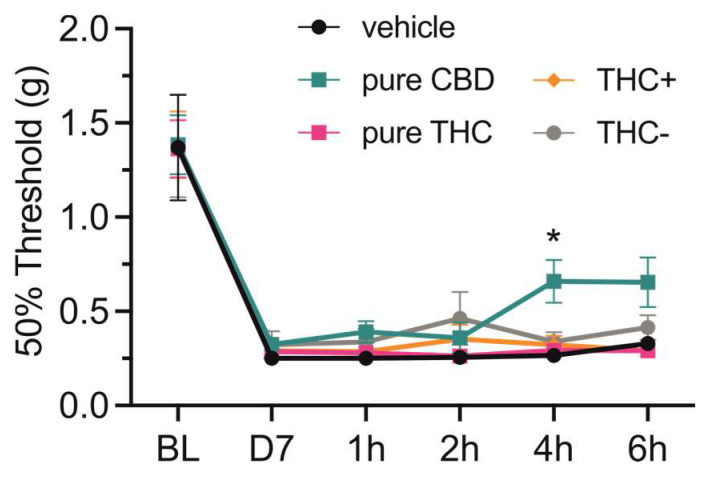
Effect of single-dose oral administration of cannabis extracts and pure cannabinoid compounds on mechanical thresholds in neuropathic mice. Mechanical paw-withdrawal thresholds of neuropathic pain mice were determined using von Frey filaments at baseline (BL), on day 7 after surgery (D7), as well as 1, 2, 4 and 6 h after single-dose oral administration of vehicle (i.e., sesame oil), 20 mg/kg pure CBD and THC compounds, 40 mg/kg THC+ extract and 1560 mg/kg THC- extract. Data are represented as mean ± SEM, *n* = 10 per treatment group. * *p* < 0.05, 2-way ANOVA followed by Dunnett’s post hoc test.

Finally, we tested if oral application of the different cannabis formulations or pure compounds would lead to different analgesic effects. For this, mice that underwent spared nerve injury (SNI) surgery were tested for mechanical hypersensitivity by mechanical threshold determination using von Frey filaments. All mice showed strong mechanical hypersensitivity seven days after surgery, and while a single oral application of vehicle (i.e., sesame oil), THC+ and THC- extract as well as pure THC compound did not affect mechanical withdrawal thresholds, application of CBD alleviated mechanical hypersensitivity 4 h after administration (2-way repeated measures ANOVA followed by Dunnett’s multiple comparisons test, 4 h—vehicle vs. pure CBD, *p* = 0.022; Figure 4).

## 4. Discussion

The current study aimed to provide first insights into whether oral administration of medical marijuana extracts could offer benefits for treatments of patients suffering from pain disorders. We report on the composition and presence of less well investigated phytocannabinoids in *Cannabis sativa* extracts and provide first results supporting a faster rise in THC levels already at one hour of administration in plasma and brain, but not in the spinal cord from a THC+ extract. These findings were also reflected by the trajectories of the psychoactive metabolite OH-THC, and this supports oral administration as a possible route for cannabinoid intake for patients suffering from pain disorders. Surprisingly, CBD but not THC alleviated signatures of mechanical hypersensitivity 4 h after administration.

Cannabis products have been tested for various routes of administration and delivery forms in order to enhance their therapeutic effect by increasing the bioavailability of principle compounds CBD and THC. Oral administration of medical marijuana is the prevalent route of administration recommended due to easy administration, prolonged drug action, and reduced toxicity [13,32]. Similar to previous studies in humans and rats, we found that THC reached its peak concentration in mouse plasma, serum or brain approximatively two hours after oral ingestion [11,33,34]. In addition, our studies provide the first data to fill the knowledge gap on tissue distribution at multiple time points following oral administration. The pharmacokinetics of THC when administered as an extract was markedly different from pure THC delivered at an equivalent dose. Specifically, THC peak plasma concentrations were reached substantially faster when administered as a THC+ extract and this may be associated with phytocannabinoids or even the presence of CBD, which is known to affect THC bioavailability [35]. Alterations of gut motility, secretion and resorption may further contribute to this observation [36,37]. Given that pure THC was administered at doses equivalent to that in the THC+ extract, other bioactive substances present in the extract, such as CBC, CBD, CBDV, CBG, CBN and THCV may account for the faster bioavailability of THC by altering its metabolism or distribution [38]. Another possibility may be that CBD and CBN act as inhibitors of enzymes of the cytochrome P450 (CYP) complex resulting in inhibition of THC metabolism in the liver [39,40].

The central nervous system (CNS) is affected by major disorders targeted by cannabinoid therapeutic interventions, such as neurodegeneration, pain and mood disorders and physiochemical properties determine drug penetration into the CNS [41,42]. The presence of other cannabinoids in THC+ extract might support THC bioavailability not only in plasma but also sustained elevated levels in the CNS, which may result in prolonged biological effects. This is also mirrored by the kinetics of THC metabolites OH-THC and COOH-THC. In plasma, both COOH-THC and OH-THC were present for all three THC formulations, whereas OH-THC was the predominant form in the brain. Considering that OH-THC induces physiological effects on its own [33], it may contribute to the therapeutic benefits of cannabinoids. However, there is no difference in OH-THC concentration following co-administration of THC and CBD [11], suggesting that CBD does not affect THC metabolism. However, in our study, the ratio between the bioactive substances of the extract is markedly different and the combination of cannabinoids might interfere with THC metabolism. CBD acts as a neuroprotectant and anti-inflammatory agent with positive safety profiles [43,44]. Our results are in accordance with an earlier study showing that CBD levels continue to rise four hours after administration and a brain–plasma ratio of 3.54 is reported when CBD is orally given to mice [45].

Surprisingly, our behavior data did not match our pharmacokinetic profiles. Neither orally administered THC+ extract nor pure THC alleviated mechanical hypersensitivity induced by nerve injury. These results challenge our understanding on drug dosing, since oral THC at 1 mg/kg or 17.8 mg/kg and intraperitoneal injection at 1.0–4.0 mg/kg or 30 mg/kg had significant analgesic effects [46,47,48], which, however, could be due to different pain models and vehicles used. A significant decrease in mechanical hypersensitivity four hours after administration was only observed for CBD and this was consistent with CBD concentrations measured in the brain and spinal cord, as well as in previous reports [24,48,49].

Our pilot evaluation suggests that unknown *Cannabis sativa* components appear to affect the bioavailability of psychoactive THC. The data support the necessity of further research aiming at a more precise administration of cannabinoid-based edibles and more precise recommendations for *Cannabis sativa* over the counter drugs [50]. In addition, variations in extract composition resulting from differences in plant strains and treatments of the harvested material and uncontrolled degradation of cannabinoids by oxygen, light or heating may be problematic. Our current data favor CBD as an analgesic since less psychoactive unwanted effects may be expected [51]. Further investigations exploring larger cohort sizes, different therapeutic windows, oral extracts versus pure compounds and clearly defined medical conditions are important to tailor analgesic cannabinoid therapies precisely to the patients’ needs.

## Figures and Tables

**Table 1:** LC-MS/MS mass transition and potentials.

Substance	MRM-Transition ^1^	Dwell Time	DP ^2^	CE ^3^	CXP ^4^	EP ^5^
Δ9-THC and CBD (Quantifier)	315.0 → 193.1	30 msec	60	30	10	10
Δ9-THC and CBD (Qualifier)	315.0 → 259.0	10 msec	60	30	10	10
D3-THC (Quantifier)	318.2 → 196.1	30 msec	60	30	10	10
D3-THC (Qualifier)	318.2 → 123.0	10 msec	60	30	10	10
OH-THC (Quantifier)	331.2 → 313.1	30 msec	60	30	10	10
OH-THC (Qualifier)	331.2 → 193.1	10 msec	60	30	10	10
COOH-THC (Quantifier)	345.2 → 299.2	30 msec	60	30	10	10
COOH-THC (Qualifier)	345.2 → 193.1	10 msec	60	30	10	10

^1^ MRM, multiple reaction monitoring; ^2^ DP, declustering potential; ^3^ CE, collision energy; ^4^ CXP, collision cell exit potential; ^5^ EP, entrance potential.

**Table 2:** Quantification of cannabinoids and flavonoids in THC^+^ extract.

Analyte	C ^1^ Per Sample [ng/mL]	Mean C [ng/mL] ^2^	SEM	C of Analysed Extract [μg/mL]	ng Analyte/ μg Extract	% [m/m]
CBC	247.5	247.40	3.44	40	6.19	0.62
	241.4					
	253.3					
CBD	63.30	63.08	1.04	40	1.58	0.16
	64.77					
	61.18					
CBDA	<LLOQ	<LLOQ ^3^	N/A ^4^	N/A	N/A	N/A
	<LLOQ					
	<LLOQ					
CBDV	38.35	37.46	2.49	400	0.09	0.01
	32.77					
	41.26					
CBG	297.2	300.37	2.16	40	7.51	0.75
	304.5					
	299.4					
CBN	2343	2677.00	175.60	40	66.93	6.69
	2750					
	2938					
THCV	110.4	90.95	12.39	40	2.27	0.23
	94.52					
	67.92					
THCA-A	170	161.33	6.55	400	0.4	0.04
	165.5					
	148.5					
Δ9-THC	1890	2043.33	79.90	4	510.83	51.08
	2081					
	2159					
API	<LLOQ	<LLOQ	N/A	N/A	N/A	N/A
	<LLOQ					
	<LLOQ					
LUT	<LLOQ	<LLOQ	N/A	N/A	N/A	N/A
	<LLOQ					
	<LLOQ					

^1^ C, concentration; ^2^ mean concentration of sample triplicates; the reported concentrations refer to the lowest extract concentration providing an analyte concentration within the calibration range; ^3^ LLOQ, lower limit of quantification; <LLOQ: no analyte concentration within the calibration range could be found in any of the analysed extract concentrations; ^4^ N/A, not available.

**Table 3:** Quantification of cannabinoids and flavonoids in THC- extract.

Analyte	C ^1^ Per Sample [ng/mL]	Mean C [ng/mL] ^2^	SEM	C of Analysed Extract [μg/mL]	ng Analyte/ μg Extract	% [m/m]
CBC	<LLOQ	<LLOQ ^3^	N/A ^4^	N/A	N/A	N/A
	<LLOQ					
	<LLOQ					
CBD	<LLOQ	<LLOQ	N/A	N/A	N/A	N/A
	<LLOQ					
	<LLOQ					
CBDA	16.19	16.16	0.04	400	0.04	0.004
	16.20					
	16.09					
CBDV	<LLOQ	<LLOQ	N/A	N/A	N/A	N/A
	<LLOQ					
	<LLOQ					
CBG	62.56	57.25	2.68	400	0.14	0.014
	55.19					
	54.01					
CBN	30.61	29.66	0.55	40	0.74	0.07
	29.66					
	28.72					
THCV	<LLOQ	<LLOQ	N/A	N/A	N/A	N/A
	<LLOQ					
	<LLOQ					
THCA-A	216.8	213.33	2.89	40	5.33	0.53
	215.6					
	207.6					
Δ9-THC	502.6	504.97	1.89	400	1.26	0.13
	508.7					
	503.6					
API	<LLOQ	<LLOQ	N/A	N/A	N/A	N/A
	<LLOQ					
	<LLOQ					
LUT	17.38	18.41	0.70	400	0.05	0.005
	19.74					
	18.11					

^1^ C, concentration; ^2^ mean concentration of sample triplicates; the reported concentrations refer to the lowest extract concentration providing an analyte concentration within the calibration range; ^3^ LLOQ, lower limit of quantification; <LLOQ: no analyte concentration within the calibration range could be found in any of the analysed extract concentrations; ^4^ N/A, not available.

## Data Availability

The data that support the findings of this study are available on request from the corresponding authors.
